# Optogenetic Neuronal Silencing in *Drosophila* during Visual Processing

**DOI:** 10.1038/s41598-017-14076-7

**Published:** 2017-10-23

**Authors:** Alex S. Mauss, Christian Busch, Alexander Borst

**Affiliations:** Max-Planck-Institute of Neurobiology, Martinsried, 82152 Germany

## Abstract

Optogenetic channels and ion pumps have become indispensable tools in neuroscience to manipulate neuronal activity and thus to establish synaptic connectivity and behavioral causality. Inhibitory channels are particularly advantageous to explore signal processing in neural circuits since they permit the functional removal of selected neurons on a trial-by-trial basis. However, applying these tools to study the visual system poses a considerable challenge because the illumination required for their activation usually also stimulates photoreceptors substantially, precluding the simultaneous probing of visual responses. Here, we explore the utility of the recently discovered anion channelrhodopsins GtACR1 and GtACR2 for application in the visual system of *Drosophila*. We first characterized their properties using a larval crawling assay. We further obtained whole-cell recordings from cells expressing GtACR1, which mediated strong and light-sensitive photocurrents. Finally, using physiological recordings and a behavioral readout, we demonstrate that GtACR1 enables the fast and reversible silencing of genetically targeted neurons within circuits engaged in visual processing.

## Introduction

Genetically expressed optogenetic ion channels and pumps confer light sensitivity to neurons of interest, allowing to control their activity on demand^1,2^. Such techniques have become powerful means to establish neuronal connectivity as well as causal relationships between neuronal activity and behavior. Remote control of neuronal activity by light has many advantages: it is fast, reversible, easy to parameterize and applicable in intact behaving animals. However, it poses challenges for studies in visual systems, since here endogenous light-sensing cells, the photoreceptors, are also activated by light required for optogenetic control. This can lead to prominent visual artifacts or, in extreme cases, even blinding. This disadvantage is particularly prevalent in the fly optic lobe, which has otherwise become a paradigmatic example for visual processing due to identified neurons and large numbers of selective driver lines for their visualization and manipulation^3,4^.

Ignoring visual artifacts, or indeed performing experiments in genetically blinded flies, depolarizing optogenetic channels have been nonetheless very useful to infer connectivity and behavioral roles upon selective neuronal activation^5–9^. In principle, selective activation should be possible in conjunction with simultaneous probing of visual circuit function. For instance, red-shifted CsChrimson^10^ or bistable Channelrhodopsin^5,11,12^ allow the spectral or temporal separation, respectively, of transgenic and endogenous rhodopsin activation. In contrast, hyperpolarizing tools are much less abundant and usually require strong illumination in spectral ranges incompatible with visual stimulation. Thus, there is a strong necessity for suitable hyperpolarizing channels, for instance, to probe the full transmission range of graded synaptic connections or to silence genetically identified neurons on demand while circuit function is being read out.

Recently, anion channelrhodopsins (ACRs) have been discovered in the cryptophyte algae species *Guillardia theta* ^13^ (GtACR1 and GtACR2). These channels are promising versatile inhibitory tools since they impart strong light-gated chloride conductance, which is much more light-sensitive than, for instance, the Halorhodopsin class of chloride pumps^14,15^. Particularly GtACR1 is of interest for applications in the fly visual system, since its activation spectrum is shifted towards longer wavelengths with respect to five of the six *Drosophila* rhodopsins (except rhodopsin 6), and in particular the main photopigment rhodopsin 1^16–18^. A recent study has demonstrated the utility of GtACR1 and GtACR2 for fast and reversible neuronal silencing in behaving flies^19^. Here, we first confirmed these findings in a *Drosophila* larval crawling assay. Since intracellular electrophysiology data on GtACRs is not yet available in flies, we performed whole-cell patch-clamp recordings in the adult *Drosophila* optic lobe. We thus obtained response curves revealing strong light-sensitive hyperpolarization mediated by GtACR1. We then explored GtACR1 utility together with electrophysiological and behavioral readouts for probing visual function. Our results demonstrate that optogenetics via GtACR1 permits selective, fast and reversible neuronal silencing in visually active circuits.

## Results

The recently discovered anion channelrhodopsins GtACR1 and GtACR2^13^ have been shown to be suitable for silencing genetically targeted neurons in intact flies with remarkably low light requirements^19^. Here, we tested their utility for silencing neurons of the optic lobe in conjunction with visual stimulation. To be able to express these channels cell-specifically in *Drosophila* using existing Gal4 lines we cloned the two EYFP-tagged coding regions into the UAS expression vector pJFRC7^20^. With the resulting vectors we generated genomic insertions in defined chromosomal locations using phiC31 integrase^21^ to obtain UAS-GtACR1-EYFP and UAS-GtACR2-EYFP flies (landing sites attP40 on 2^nd^ and VK00005 on 3^rd^ chromosome).

### Assessing the efficacy of optogenetic tools using larval crawling as a behavioral readout

**Figure 1:**
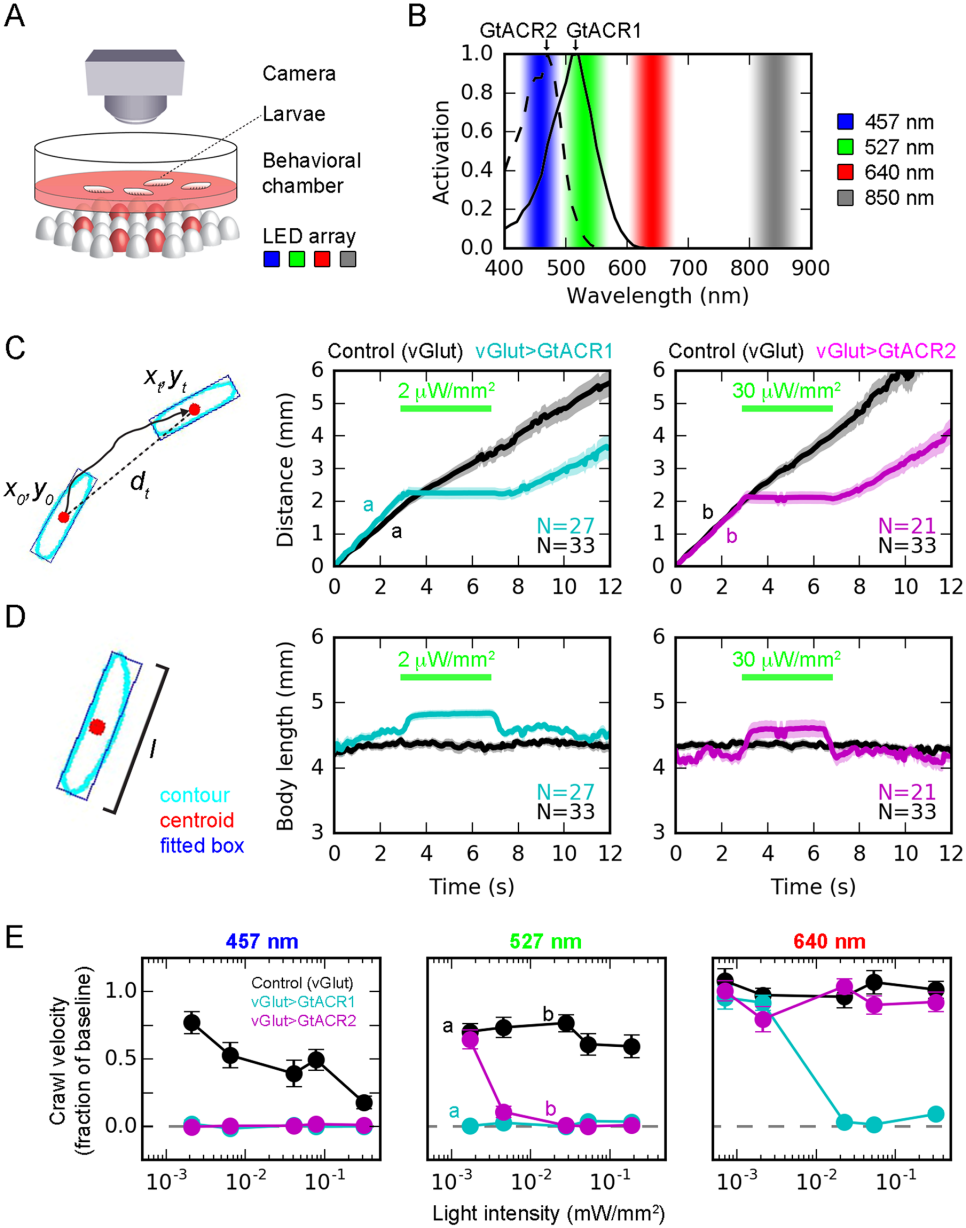
Characterization of GtACR1 and GtACR2 using a larval crawling assay. (**A**) Larvae were released in an agarose-coated petri dish and their crawling activity video-taped from above. Infra-red background illumination was provided by LED arrays emitting 850 nm light from below. Furthermore, three other LED arrays below emitted 457, 527 and 640 nm illumination for GtACR activation. Only one illumination was used for optogenetic stimulation at a time, here exemplified in red. (**B**) Relative activation spectra of GtACR1 and GtACR2, replotted from ref.^13^, with LED illumination used in the larval crawling assay indicated. (**C**) To quantify crawling activity, the measured centroid positions (red dots) were plotted as covered distance over time. The crawling activity of control larvae (vGlut-Gal4 only, no GtACR expression; black trace) was only mildly affected by illumination (527 nm at indicated intensity). In contrast, larvae with GtACR1 (cyan) or GtACR2 (magenta) expression in glutamatergic neurons (including motorneurons) seized crawling immediately with onset of light. Offset of illumination restored crawling activity. Traces labeled with a and b refer to data points in (**E)**. (**D**) As another behavioral parameter, body length was quantified by fitting a rectangle to each larva contour and measuring its length. Upon illumination, only larvae with GtACR expression in glutamatergic neurons (vGlut-GtACR) elongated, in agreement with a relaxation of the body wall musculature due to GtACR-mediated motorneuron silencing. (**E**) Crawling activity (fraction of baseline) for illumination with three different wavelengths as a function of light intensity. Letters a and b indicate data points for which example traces are displayed in (**C)**. All data are presented as mean ± standard error of the mean.

In order to assess the efficacy of optogenetic tools in *Drosophila* in a first approach, we devised a high-throughput larval crawling assay. We expressed GtACR1 and GtACR2 using vGlut-Gal4 driving expression in glutamatergic neurons including motorneurons and reasoned that silencing those should manifest in easily quantifiable reduction in crawling activity. For behavioral analysis, we obtained video data from batches of 3^rd^ instar larvae simultaneously crawling in a petridish (batch size ~10; for each experiment, on average 26.6 ± 6.2 (S.D.) larvae were tracked) (Fig. 1A). The dish was illuminated from below by LED arrays of different wavelengths: 850 nm as background illumination for image capture and 457 nm (blue), 527 nm (green) and 640 nm (red) for optogenetic stimulation, guided by the two published activation spectra of GtACR1 and GtACR2^13^ (Fig. 1B). Behavioral parameters were extracted offline from video data in an automated fashion (see Methods). We defined locomotor activity as the covered distance over time (Fig. 1C). The example traces show strong effects of illuminating vGlut > GtACR-expressing larvae in that they immediately cease to crawl. This effect is fully reversible and illuminated larvae resume crawling shortly (~1 s) after light offset. We also used body length as another behavioral measure (Fig. 1D). Exposing vGlut-GtACR-larvae with light increases body length as abruptly as it stops crawling, in line with a presumed relaxation of body wall musculature due to motor neuron inactivation.

Next, we quantified the effects of GtACR1 and GtACR2 as a function of wavelength and intensity (Fig. 1E). These experiments revealed different light requirements of GtACR1 and GtACR2, in agreement with their different activation spectra^13^. GtACR1 activation caused a full reduction of crawling activity for both blue and green light at all intensities tested (2–300 μW/mm^2^), while red light required intensities of at least 20 μW/mm^2^. GtACR2 was most effective with blue light, showing full crawling suppression. However, green light required > 5 μW/mm^2^ and red light did not produce any crawling phenotype for intensities below 300 μW/mm^2^. To conclude, our data are in agreement with the literature^13,19^. Particularly GtACR1 with an activation peak shifted relative to rhodopsin 1 seemed to be a promising candidate for selective neuronal silencing within visually active circuits. In the following, we therefore focus on the characterization of GtACR1 in the *Drosophila* optic lobe.

### Light requirements of neuronal hyperpolarization by GtACR1

**Figure 2:**
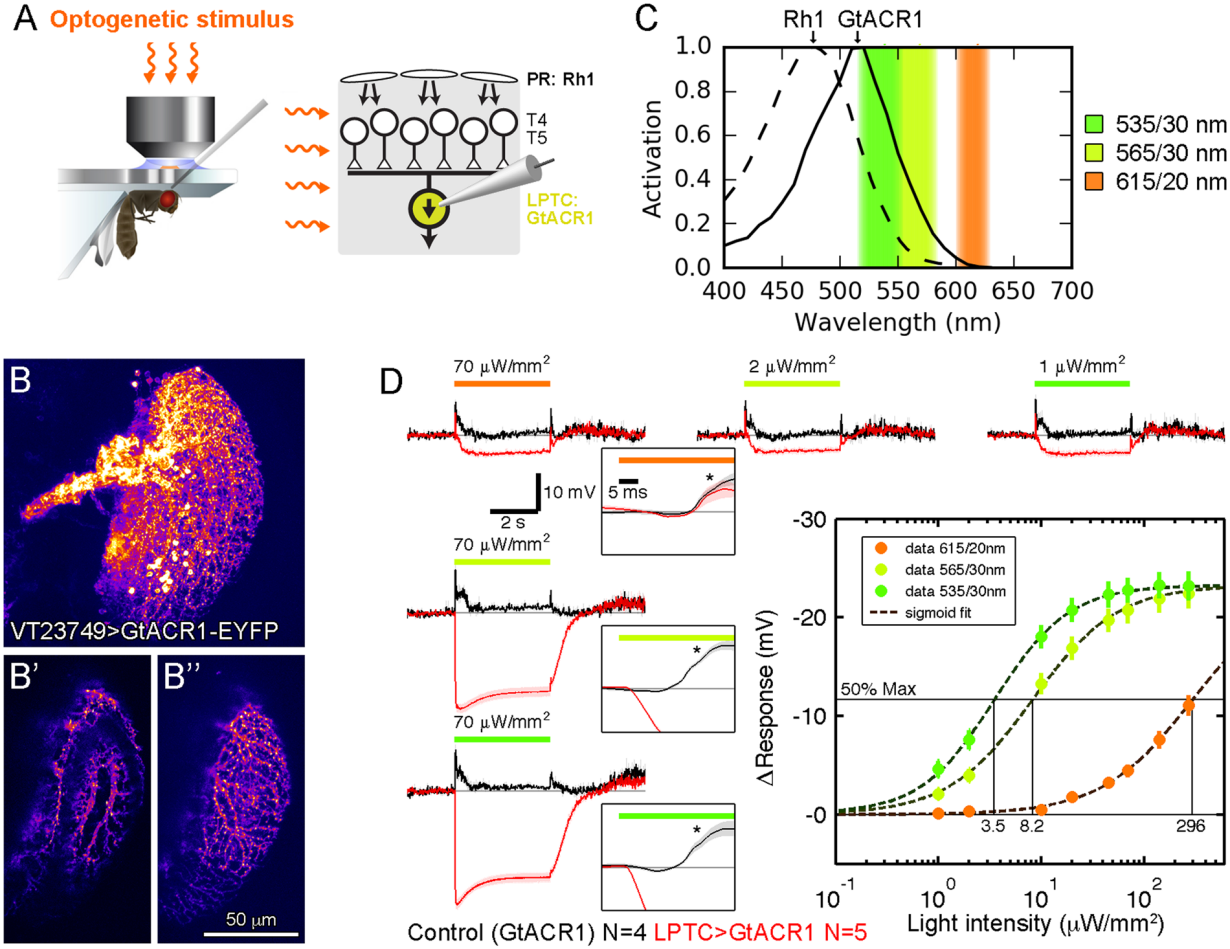
Characterization of GtACR1 in lobula plate tangential cells by whole-cell patch-clamp recordings. (**A**) Illustration of preparation for tangential cell recordings (left schematic adapted with permission from ref.^22^). Lobula plate tangential cells (LPTCs) receive direction-selective visual input from T4/T5 neurons, three synapses downstream of photoreceptors (PR). Illumination for GtACR1 activation in tangential cells is conveyed to the brain via the epi-fluorescent light path of the microscope. (**B-B”**) Confocal images showing expression of GtACR1-EYFP in tangential cells. **B** depicts a maximal projection and **B’**, **B”** show projections from z-subsections highlighting individual dendritic branches. (**C**) Relative activation spectra of photoreceptor rhodopsin 1 (replotted from ref.^17^) and transgenically expressed GtACR1 (replotted from ref.^13^). Center illumination wavelengths (e.g. 615 nm) and bandwidths (e.g. 20 nm) used for the following experiments are indicated. (**D**) GtACR1-expressing tangential cell responses (membrane potential) to illumination of indicated wavelengths and intensities over time, averaged across 8 trials and N cells. Different wavelengths of similar intensity cause hyperpolarizations of different amplitudes (traces on the left). The same hyperpolarization in cells can be achieved with different wavelengths at different intensities (traces on top). Voltage traces with an expanded time axis are shown in the insets, showing a ~15 ms delayed depolarizing visual response (asterisk) that is replaced by short-latency (2–3 ms) GtACR1-mediated hyperpolarization using 535 and 565 nm illumination (red trace). The responses are quantified as the baseline-subtracted time-averaged potential during the steady-state (3–4 s after illumination onset minus 1–0 s before illumination onset). For each wavelength, sigmoid functions were fitted to the response amplitudes to obtain the light intensities required to reach 50% of the maximal response. Data are presented as mean ± standard error of the mean.

GtACRs are expected to hyperpolarize neurons, depending on the chloride reversal potential, which can be rigorously addressed only by intracellular electrophysiological recordings. In order to fully characterize mode of action and light requirements, we aimed to directly measure the GtACR1-mediated physiological effects in single neurons. Tangential cells of the lobula plate lend themselves well for this purpose since whole-cell patch-clamp recordings can be readily obtained from their large cell bodies^22^ (Fig. 2A). Tangential cells characteristically respond with graded potential changes of about 5–15 mV, depending on the stimulus, to visual wide-field motion: depolarization in response to the preferred direction and hyperpolarization in response to the opposite or null direction^22^. Using a selective driver line, we expressed GtACR1 in tangential cells (Fig. 2B-B”). To activate GtACR1, we passed light from a Xenon arc lamp through optic band pass filters (resulting wavelengths relative to Rh1 and GtACR1 shown in Fig. 2C) and delivered it to the preparation via the epifluorescent light path of the microscope. Illumination of brains in control flies without GtACR1 expression resulted in transient ON and OFF tangential cell voltage deflections, in line with the light reaching the photoreceptors^7^, but little tonic changes. In stark contrast, GtACR1-expressing tangential cells responded with strong hyperpolarization of up to 22 mV on average (Fig. 2D), which is roughly twice the amplitude of robust visually evoked null direction inhibition^22^. The hyperpolarization onset latency (see small insets in Fig. 2D, red trace) was in the range of 2–3 ms and therefore much faster than the one of the visual ON transient in the control condition (~15 ms). In line with GtACR1’s spectral response peak, light of 535 nm wavelength was most effective and 615 nm light had to be of considerable higher intensity to reach the same effects. We quantified responses for each tested wavelength as a function of light intensity and fitted sigmoidal functions to the responses. Thus, we obtained light intensities at 50% maximal hyperpolarization of 3.5, 8.2 and 296 μW/mm^2^ for 535, 565 and 615 nm, respectively (Fig. 2D).

### Neuronal silencing using GtACR1 is compatible with simultaneous visual stimulation in a physiological preparation

**Figure 3:**
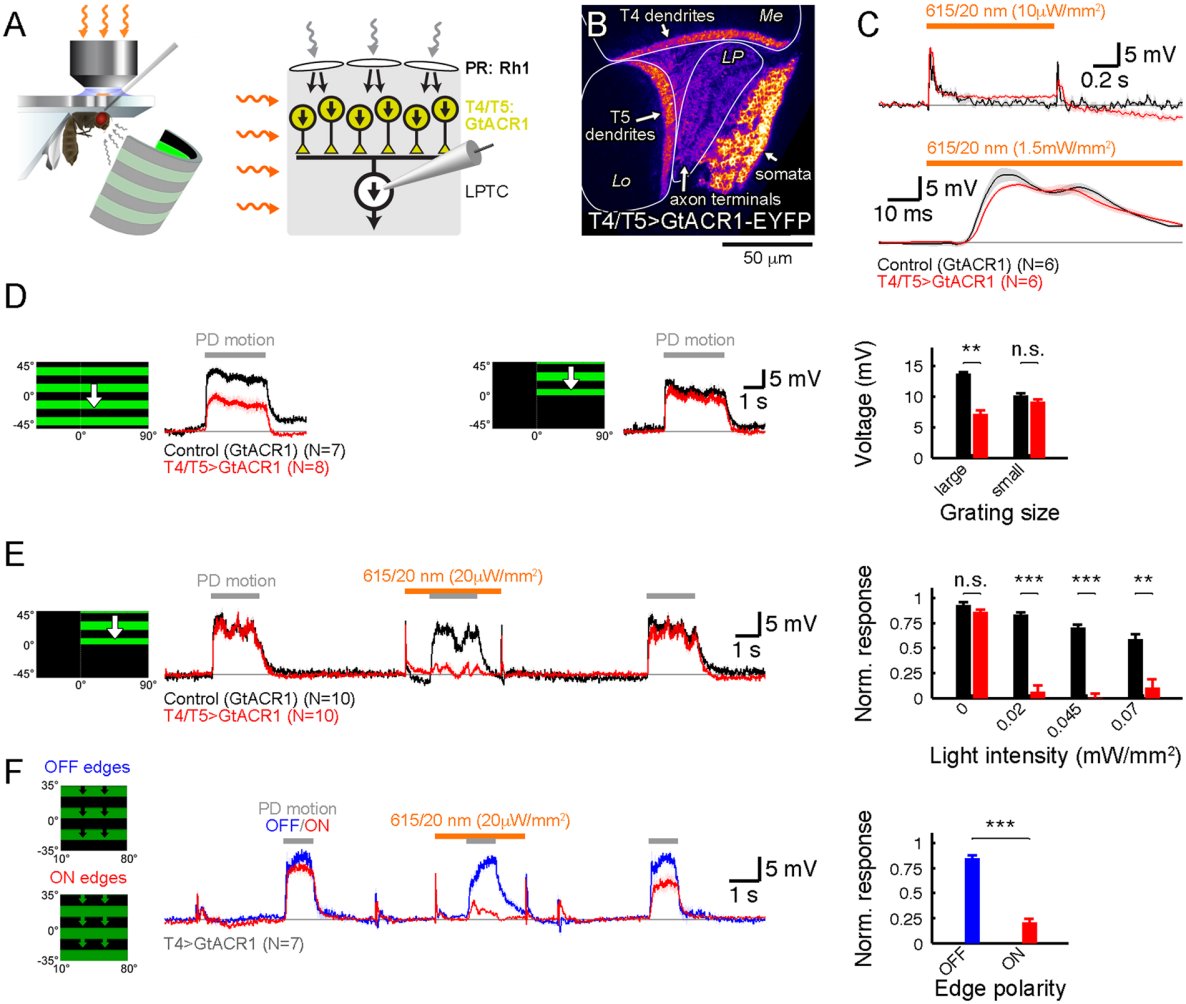
Using GtACR1 for optogenetic silencing of visual motion inputs to tangential cells. (**A**) Illustration of preparation for tangential cell recordings with GtACR1 expression in upstream direction-selective T4/T5 neurons (left schematic adapted with permission from ref.^22^). (**B**) Confocal image showing expression of GtACR1-EYFP in T4/T5 neurons in a horizontal cross section. Me, medulla; Lo, lobula; LP, lobula plate. (**C**) Tangential cell responses in control (black traces) and T4/T5 > GtACR1 flies (red traces) to 615 nm illumination of indicated intensities. Note the different time scales. (**D**) Tangential cell responses in control (black traces) and T4/T5 > GtACR1 flies (red traces) to gratings of different sizes moving in the preferred direction. For the large pattern, cells in T4/T5 > GtACR1 flies show a reduced average response compared to control flies, presumably due to GtACR1 activation by the visual stimulus. Quantifications represent time-averaged and baseline-subtracted membrane potentials. (**E**) Tangential cell responses in control (black traces) and T4/T5 > GtACR1 flies (red traces) to combined visual and optogenetic stimulation. Visual stimuli were presented three times per trial and the second stimulation combined with 615 nm illumination (average voltage traces shown for 20 μW/mm^2^). Responses in control flies become progressively more reduced at increasing illumination intensities yet still reach ~50% at the highest intensity. In contrast, responses in T4/T5 > GtACR1 flies are eliminated already by weak illumination. For quantification, time-averaged membrane potentials were baseline-subtracted for the first and second visual stimulus. A normalized response was obtained by dividing the second by the first response. (**F**) Tangential cell responses to moving ON (red) and OFF edges (blue) in the same individuals expressing GtACR1 in ON-selective T4 cells. The stimulus is presented three times per trial and the second time combined with 615 nm light illumination. The OFF response is comparable to wild type while the ON response is almost absent. The third visual ON response is slightly reduced for unknown reasons. Quantification as in (**E)**. Traces in **C**–**F** represent the membrane potential averaged across 4 trials and N cells. All data are shown as mean ± standard error of the mean. A two-tailed Wilcoxon ranksum test was performed to establish statistical significance: n.s., not significant; **p < 0.01; ***p < 0.001.

The light requirements of GtACR1 in terms of wavelength and intensity seemed potentially suitable to silence neurons in the visual pathway without strongly activating photoreceptors. To test this, we took advantage of the fact that the visual pathways impinging on lobula plate tangential cells are characterized in exquisite detail^3^. Tangential cells receive direct cholinergic input from arrays of local direction-selective T4/T5 neurons, giving rise to preferred direction excitation^7,23,24^. In addition, tangential cells receive indirect inhibitory input from oppositely tuned T4/T5 cells via glutamatergic interneurons, causing hyperpolarization during null direction motion^6^. Therefore, all visual motion responses in tangential cells require T4/T5 cell activity, providing an ideal test bed for combined visual stimulation and optogenetic silencing (Fig. 3A).

In an initial set of experiments, we explored the suitability of different wavelengths for combined optogenetic and visual stimulation. To this end, we recorded from tangential cells in control flies (no GtACR1 expression) and illuminated the brain with 535, 565 and 615 nm light previously established to produce the same GtACR1-mediated hyperpolarization (1, 2 and 70 μW/mm^2^, respectively; see Fig. 2D). Simultaneously probed visual responses were indistinguishable for 565 and 615 nm but reduced for 535 nm (data not shown). This unfavorable effect of 535 nm light is potentially caused by wavelength-dependent relative differences in absorption by Rhodopsin1 or its activated metarhodopsin state and GtACR1. In the following electrophysiological experiments, we therefore used 615 nm light, although a slightly shorter wavelength such as 565 nm also seemed suitable.

To assess direct effects of T4/T5 cell hyperpolarization in absence of visual stimulation, we recorded from tangential cells in control flies carrying only the GtACR1 transgene without the driver, hence termed “Control (GtACR1)”, and in flies expressing GtACR1 in presynaptic T4/T5 cells, termed “T4/T5 > GtACR1” (Fig. 3B). When illuminating the brain with 615 nm light, expected ON and OFF transients due to photoreceptor activation became apparent in both conditions, with little tonic effects on the membrane potential (Fig. 3C). The time course of the ON transient with an onset latency of 15 ms was virtually indistinguishable between both genotypes. Thus, it appears that only positive T4/T5 signals are transmitted to downstream tangential cells^7^.

Next, to test whether GtACR1-expressing T4/T5 cells retain their normal visual function, we stimulated control and T4/T5 > GtACR1 flies visually with moving gratings, while recording from tangential cells. For large patterns, we measured reduced visual responses in the latter experimental group (Fig. 3D, left traces and quantification). Since GtACR1 is rather light sensitive and the visual stimulus arena emits 565 nm light, well suited to activate GtACR1, we reasoned that the ambient light might already be sufficient to partially silence GtACR1-expressing T4/T5 cells. To test this, we reduced total luminance from the arena by decreasing the pattern size. Now, responses in the two genetic conditions were indistinguishable (Fig. 3D, right traces and quantification), in agreement with cross-activation of GtACR1 from arena light in the large pattern condition.

To determine whether GtACR1 is suitable to conditionally silence T4/T5 neurons in visually active circuits, we combined visual and optogenetic stimulation in control and T4/T5 > GtACR1 flies while recording from tangential cells (Fig. 3E). One stimulus sequence consisted of three identical visual stimulations (grating moving downward, i.e. in the preferred direction of the recorded cells), with the second one combined with optogenetic illumination (615 nm light of varying intensities). Control flies’ visual responses became progressively reduced with increasing light intensities (due to interference of the optogenetic illumination with photoreception) but were still robustly detectable at 70 μW/mm^2^ (Fig. 3E). In T4/T5 > GtACR1 flies however, visual responses during illumination were almost entirely absent at light intensities of 20 μW/mm^2^ and above. Importantly, the first and third visual response in each trial were not different between T4/T5 > GtACR1 and control flies, demonstrating the normal function of GtACR1-expressing neurons immediately before and after exposure to optogenetic illumination.

Like in the vertebrate retina, visual motion processing in flies is split into an ON- and an OFF-pathway^3,25^ with T4 cells being the first direction-selective neurons in the ON- and T5 cells the first ones in the OFF-pathway^23,26–28^. To rule out any confounding effects on visual sensitivity due to genetic background we performed another set of experiments with an internal control for visual function. To this end, we expressed GtACR1 in T4 cells only and stimulated flies with moving ON or OFF edges (Fig. 3F). Importantly, moving decrements of light (OFF edges) are processed in the parallel pathway by T5 cells, which do not express GtACR1 in this experiment and should thus retain their normal function. In downstream tangential cells, ON and OFF responses should thus be differentially affected by optogenetic illumination in the same animal. Indeed, OFF edge responses were hardly reduced by illumination, while ON edge responses were almost completely abolished (Fig. 3F). We also noted a slight average decrease in the third visual response amplitude immediately after optogenetic stimulation. However, this response was still markedly larger than the preceding one during optogenetic stimulation (second visual response) and fully recovered until the following trial. Taken together, our experiments unequivocally demonstrate the selective optogenetic silencing in the visual circuit while leaving vision functional.

### Neuronal silencing using GtACR1 is compatible with simultaneous visual stimulation in intact behaving animals

**Figure 4:**
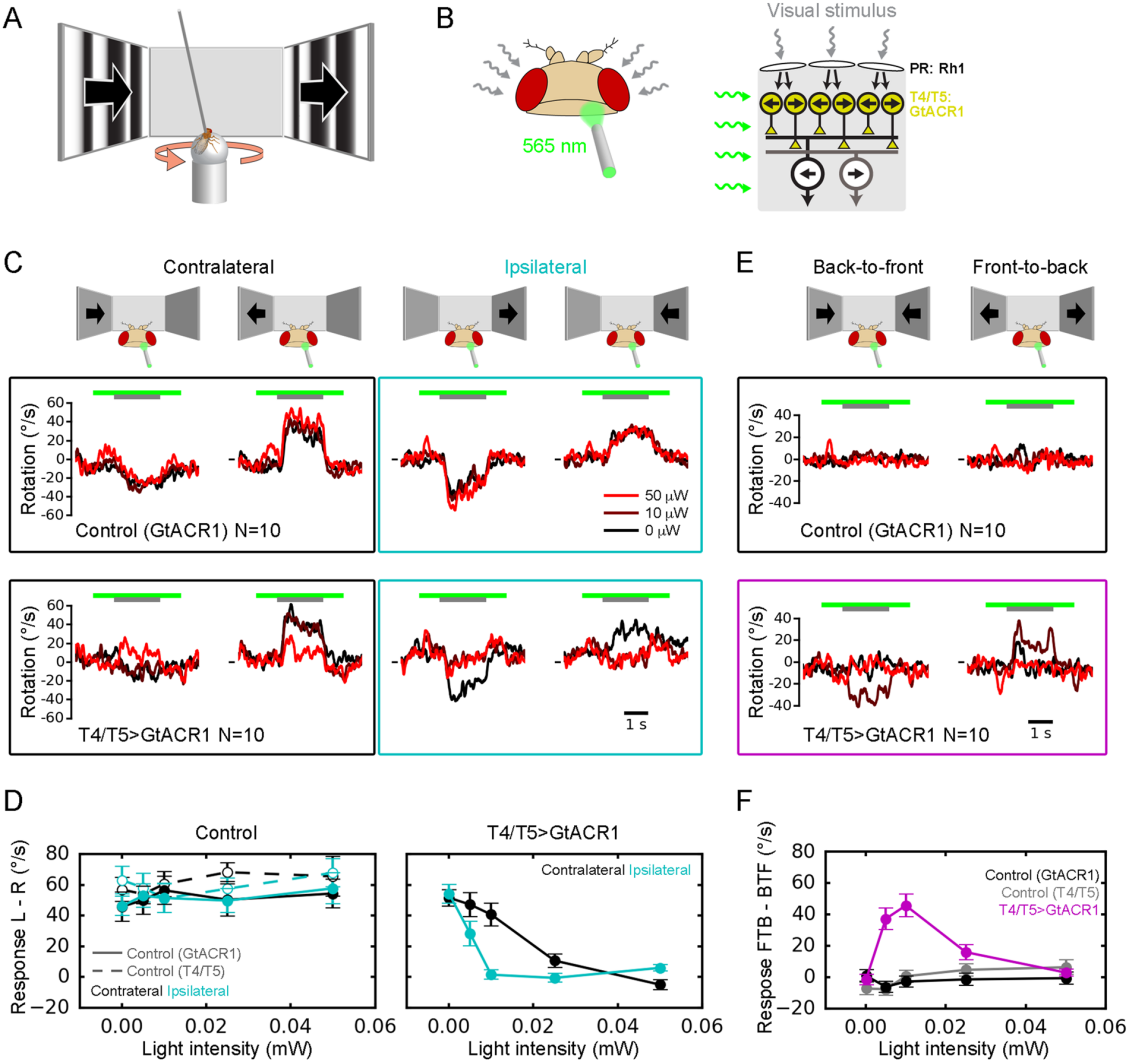
Using GtACR1 for optogenetic silencing of visual motion signals underlying the optomotor response. (**A**) Schematic illustrating the behavioral optomotor assay. A tethered fly is walking on an air-suspended ball whose rotation is measured, allowing to obtain fly turning responses to visual motion. (**B**) To optogenetically silence visual neurons expressing GtACR1, light is focused onto a small spot (0.12 mm^2^) on the back of the fly head. (**C**) Fly turning responses (averaged across 20 trials and 10 flies) to visual motion towards left and right, presented either on the same (ipsilateral) or contralateral side of optogenetic illumination. Response traces for three illumination conditions are overlaid (0, 10 and 50 μW). Control flies (upper row) show no discernible changes in optomotor behavior due to illumination. In contrast, T4/T5 > GtACR1 flies display markedly reduced optomotor turning upon illumination (lower row), particularly in combination with ipsilateral visual stimulation (two plots on the right). The short horizontal lines in front of traces indicate zero turning. (**D**) Quantification of experiment presented in **C**, with an additional control (T4/T5 driver only, i.e without expression) and two additional optogenetic light intensities. Baseline-subtracted responses to left- and right-ward motion were combined (L–R) separately for ipsi- and contralateral stimulation. (**E**) Experiment as in **C** but with simultaneous visual motion in opposite directions on left and right side (back-to-front, shown on the left; front-to-back, shown on the right). Control flies do not display turning on average in any condition. T4/T5 > GtACR1 flies respond with turning to both visual stimuli when combined with illumination of intermediate intensities, in agreement with GtACR1-mediated unilateral motion blindness. The short horizontal lines in front of traces indicate zero turning. (**F**) Quantification of experiment presented in **E**, with an additional control (T4/T5 driver only, i.e without expression) and two additional optogenetic light intensities. Baseline-subtracted responses to front-to-back and back-to-front were combined (FTB - BTF). Data in **D** and **F** represent the mean ± standard error of the mean.

A powerful application for optogenetic tools is to control neuronal activity in intact animals, thus establishing causal relationships between neuronal activity and behavior. We wanted to test the potential of GtACR1 for silencing neuronal activity in fly visual circuits while simultaneously tracking visually controlled behavior. As a readout, we used tethered flies walking on an air-suspended ball allowing us to precisely measure their turning tendency in response to panoramic visual motion (optomotor response^29^) (Fig. 4A). Permanent blocking of T4 and T5 cells by expressing the temperature-sensitive shibire allele or tetanus toxin light chain had previously been shown to abolish the optomotor response completely and render flies motion-blind^30,31^. Again, we expressed GtACR1 in T4/T5 neurons, termed “T4/T5 > GtACR1”, and used flies with T4/T5-Gal4 driver or UAS-GtACR1 only as genetic parental controls without GtACR1 expression, termed “Control (T4/T5)” and “Control (GtACR1)”, respectively. We then measured the optomotor response in presence and absence of 565 nm light illumination of varying intensities focused onto a 0.12 mm^2^ spot to the back of the head on the right side (Fig. 4B). Since the optogenetic illumination has to penetrate the cuticle, which is expected to scatter and filter out a considerable proportion of photons, higher light intensities compared to the physiological experiments were used. Control flies exhibited turning responses similar to baseline (no illumination) upon visual stimulation up to light intensities of 50 μW, demonstrating negligible visual interference of the optogenetic light stimulus (Fig. 4C,D). T4/T5 > GtACR1 flies, however, displayed marked reduction in their turning responses upon illumination. As expected, this phenotype was more light-sensitive for visual stimulation on the same side of optogenetic illumination. Visual responses to moving patterns on the contralateral side were also reduced at higher light intensities, probably due to light scattering within the head capsule across hemispheres.

To further demonstrate selective T4/T5 silencing during visual processing, we presented flies with either back-to-front or front-to-back bilateral motion. Control and T4/T5 > GtACR1 flies without illumination showed no average turning response. This was expected, since the opposing motions on both sides as normally perceived during forward or backward translation do not elicit a directed turn (Fig. 4E,F). However, with increasing illumination intensities (5–10 μW), T4/T5 > GtACR1 flies on average increased their turning with the motion direction presented on the contralateral side. This finding is in agreement with optogenetically mediated, ipsilateral motion blindness due to the silencing of T4/T5 cells. At yet higher intensities, average turning decreased to baseline, again most likely due to the contralateral spread of light.

## Discussion

Targeting light-gated hyperpolarizing ion channels and pumps to genetically defined neuron types is a powerful means to control their activity on demand^1,2^. However, applying this approach to visual circuits is highly problematic because the required light typically also stimulates endogenous light-sensing photoreceptors, often even beyond saturation. Here, we demonstrate in *Drosophila* that the recently discovered anion channelrhodopsin GtACR1 has the necessary properties to enable optogenetic neuronal silencing in active visual circuits.

Optogenetic control over neuronal activity within visual circuits essentially requires independent gating of endogenous and transgenic rhodopsins by light. Primary visual circuits, such as the mammalian retina or the insect optic lobe, are usually very close to photoreceptors so that spatial restriction of illumination is exceedingly difficult in translucent neural tissue. Instead, different activation spectra of rhodopsins can be exploited for independent control with different wavelengths of light, but only given suitable other properties such as sensitivity and conductance. For instance, the chloride-pumping halorhodopsins^14,15^ exhibit peak activation at ~600 nm quite separated from the main photopigment rhodopsin 1 in *Drosophila* (~480 nm^16^). However, their strong light requirements seem inadequate for leaving photoreceptors functional. The anion channel rhodopsins GtACR1 and GtACR2 in turn mediate large photocurrents that are orders of magnitude more sensitive^13^. GtACR1 is maximally activated by 515 nm light, ~30 nm apart from rhodopsin 1. Here, we have demonstrated that this spectral difference is sufficient for independent control of visually stimulated photoreceptors and GtACR1-expressing visual neurons illuminated from the back of the head. However, not surprisingly, since the activation spectra substantially overlap (Fig. 2C), illumination has to be carefully calibrated in order to keep photoreceptor activation minimal. Cross-activation can also occur in the other direction in that visual stimuli reach and partially silence GtACR1-expressing neurons, adding another parameter to control for. In support of this notion, tangential cell responses on average are reduced in flies with T4/T5 > GtACR1 expression when using the full spatial range and luminance of our visual stimulation arena (Fig. 3D).

The family of excitatory optogenetic channels has undergone considerable technical modifications which exemplify how the above-mentioned issues could be alleviated, either by molecular engineering or genomic screening: 1) slowed kinetics rendering anion channelrhodopsins switchable could be used to maintain inhibitory conductance for some time after offset of illumination^5,11,12,32^; or 2) anion channelrhodopsins with red-shifted activation spectra more separated from those of endogenous rhodopsins would greatly improve independent spectral control^10,14,32,33^. Recent work has begun to expand the family of natural and artificial anion conducting channelrhodopsins in this direction to generate variants with altered kinetics and spectral sensitivities^34,35^.

While GtACR’s are rather selective for chloride ions, two studies in rats have found surprising activating effects in axon terminals. GtACR1-expressing thalamocortical terminals exhibited neurotransmitter release and, as a consequence, evoked strong and short-latency excitatory postsynaptic currents in downstream neurons upon light onset^36^. A similar mode of action was ascribed to GtACR2, which mediated the generation of antidromic action potentials in cortical pyramidal neurons^37^. These activating effects of GtACR’s were suggested to arise by depolarized chloride reversal potentials in axon terminals. Here, in flies, we have found that tangential cells expressing GtACR1 exhibit pure hyperpolarization with a fast onset latency of 2–3 ms, as measured at the soma (Fig. 2D). Furthermore, illumination of GtACR1-expressing presynaptic T4/T5 neurons does not lead to short-latency excitatory potentials in tangential cells (Fig. 3C), as would be expected in case of transient T4/T5 activation^7^. Longer latency transients (~15 ms onset) became apparent but those are almost certainly of visual origin since they also occur in control flies without GtACR1 expression and are absent in blind flies^7^. Therefore, we consider an excitatory action of GtACR1 unlikely in flies, at least in the neuron types considered here.

In the *Drosophila* visual system and beyond, other genetic strategies are available to silence neuronal output^4,38^. Widely used temperature-sensitive dynamin (shibire-ts^39^) interferes with synaptic vesicle recycling and thus depletes chemical synaptic transmission. However, applied temperature changes may cause physiological or behavioral phenotypes and are in practice brought about only on slow time scales. This way, permissive and non-permissive conditions are rarely applied within a single experiment. While still useful in many cases, experiments with absent activity in downstream neurons as a consequence of chronic perturbation can be exceedingly difficult to interpret. For instance, 2-photon calcium imaging experiments often require some evoked neuronal activity to target the site of optical recording. The use of synaptic silencers can also be problematic because their efficacy may depend on the cell type and their molecular composition, as for instance indicated by differential phenotypic penetrance between expression of shibire and tetanus toxin light chain^40,41^. Furthermore, chemical synaptic silencers do not affect transmission across gap junctions, leaving a potential source for phenotypic underestimation. Expression of the potassium channel Kir2.1^42^ circumvents this latter issue but this tool is not inducible.

Two properties of optogenetic inhibitory ion channels can overcome the above-mentioned drawbacks of conventional neurogenetic silencers: first, they are inducible and reversible on fast time scales; and second, their mode of action by opening inhibitory conductances should be applicable to any neuron type, provided a chloride reversal potential below rest, and affect chemical and electrical synaptic output alike. Transgenically expressed GtACR1 as described here makes it possible to effectively hyperpolarize selected neurons on demand in electrophysiological and behavioral preparations without marked interference with visual perception, given a specific and sufficiently strong expressing neuronal driver line. This will be very useful to probe the requirement of identified cell types for visual and other computations by taking neuronal elements out of the circuit on a trial-by-trial basis. The efficacy is dependent on light intensity and thus tunable, allowing to establish dose-response relationships. The fact that the control and experimental conditions are provided in the same individual will greatly facilitate data interpretation and statistics. Furthermore, the temporally precise silencing of neurons could be crucial to investigate the temporal integration of signals in downstream stages. Importantly, many neurons particularly in the optic lobe transmit synaptic signals in a graded fashion. Inducible hyperpolarizing channels such as GtACRs will be very useful to probe their full transmission range and to complement optogenetic depolarization studies to characterize synaptic connectivity of candidate neurons. Finally, in order to probe behavioral causality in naturalistic contexts, one can now envisage the tantalizing possibility of switching selected neurons repeatedly off and back on again during unrestrained, visually guided behavior.

## Methods

### Flies

Flies were raised at 25 °C and 60% humidity on standard cornmeal agar medium at a 12 h light/dark cycle. The following fly strains were used: *vGlut-OK371-Gal4* (ref.^43^; glutamatergic neurons targeted in the larval crawling assay; courtesy of the Bloomington Stock Center), *VT23749-Gal4* ^*attP2*^ (lobula plate tangential cells targeted in electrophysiology; courtesy of Barry Dickson), *R42F06-Gal4* ^*attP2*^ (ref.^24^; T4/T5 cells targeted in electrophysiology; courtesy of Gerald Rubin), *R59E08-AD* ^*attP40*^ + *VT16255-DBD* ^*attP2*^ (T4 cells targeted in electrophysiology; previously unpublished Split-Gal4 line, kindly provided by Georg Ammer & Barry Dickson), *R59E08-AD* ^*attP40*^ + *R42F06-DBD* ^*attP2*^ (ref.^31^; Split-Gal4 line expressing in T4/T5 cells targeted in the optomotor behavior assay).

### Generation of transgenic GtACR lines

GtACR1-EYFP and GtACR2-EYFP coding regions were PCR-amplified from the vectors pFUGW-hGtACR1-EYFP and pFUGW-hGtACR2-EYFP (kindly provided by John Spudich, Addgene plasmids #67795 and #67877, respectively), introducing an XbaI restriction site at the 3’ end immediately after the stop codon. PCR products were then XbaI-digested and inserted via mixed sticky-blunt end ligation into the pJFRC7-20XUAS backbone (obtained by XhoI digestion, blunting via T4 DNA polymerase and XbaI digestion of pJFRC7-20XUAS-IVS-mCD8::GFP, kindly provided by Gerald Rubin, Addgene plasmid #26220). The resulting vectors pJFRC7-20XUAS-GtACR1-EYFP and pJFRC7-20XUAS-GtACR2-EYFP were sent to the Department of Genetics Fly Facility, University of Cambridge, for injection and phiC31-mediated integration^21^ into landing sites attP40 on 2^nd^ and VK00005 on 3^rd^ chromosome to obtain transgenic UAS fly strains. All experiments presented in this paper were done with 3^rd^ chromosomal VK00005 insertions. However, we have also tested 2^nd^ chromosomal attP40 *UAS-GtACR1* and *UAS-GtACR2* insertions in the larval crawling assay without detecting any discernible difference in performance (data not shown).

### Genotypes used for experiments

Figure 1: (1) *w*−*/w*− ; *vGlut-Gal4/vGlut-Gal4 ;* + (“Control (vGlut)”, no expression), (2) *w*−*/w*− ; *vGlut-Gal4/*+ ; *UAS-GtACR1-EYFP*/+ (“vGlut>GtACR1”, GtACR1 expression in glutamatergic neurons), (3) *w*−*/w*− ; *vGlut-Gal4/*+ ; *UAS-GtACR2-EYFP/*+ (“vGlut>GtACR2”, GtACR2 expression in glutamatergic neurons). Figure 2: (1) *w*−*/w*− ; + ; *UAS-GtACR1-EYFP/UAS-GtACR1-EYFP* (“Control (GtACR1)”, no expression) (2) *w*−*/w*− ; + ; *VT23749-Gal4/UAS-GtACR1-EYFP* (“LPTC>GtACR1”, GtACR1 expression in lobula plate tangential cells). Figure 3: (1) *w*−*/w*− ; + ; *UAS-GtACR1-EYFP/UAS-GtACR1-EYFP* (“Control (GtACR1)”, no expression)  (2) *w*−*/w*− ; + ; *R42F06-Gal4/UAS-GtACR1-EYFP* (“T4/T5>GtACR1”, GtACR1 expression in T4/T5 cells)  (3) *w*−/w− ; *R59E08-AD/*+ ; *VT16255-DBD/UAS-GtACR1-EYFP* (“T4>GtACR1”, GtACR1 expression in T4 cells). Figure 4: (1) w + /w+ ; + ; *UAS-GtACR1-EYFP/UAS-GtACR1-EYFP* (“Control (GtACR1)”, no expression) (2) *w*+*/w*+ ; *R59E08-AD/R59E08-AD* ; *R42F06-DBD/R42F06-DBD* (“Control (T4/T5)”, no expression) (3) *w* + */w*+ ; *R59E08-AD/* + ; *R42F06-DBD/UAS-GtACR1-EYFP* (“T4/T5>GtACR1”, GtACR1 expression in T4/T5 cells).

### Larval crawling assay

Larvae were raised in standard cornmeal agar medium supplemented with 1 mM *all-trans* retinal (ATR, R2500; Sigma Aldrich), a necessary co-factor of channelrhodopsins. 3^rd^ instar larvae were released in batches of ~10 into a petridish (diameter 3.5 cm) coated with a thin layer of 2% agarose. For each genotype, wavelength and light intensity, a minimum number of 16 and on average 26.6 ± 6.2 (S.D.) larvae were tracked.

*Optogenetic stimulation*. Larvae were exposed to illumination from blue (457 nm peak), green (527 nm peak) and red (640 nm peak) LUXEON Rebel LEDs below, controlled from Python2.7 via a bus-powered multifunction DAQ USB device (USB-6008/6009, National Instruments).

*Image capture*. Larvae were filmed from above with a PointGrey USB3.0 camera (FL3-U3-13S2M-CS) equipped with a Fujinon lens (LENS-30F2-V80CS, 2.8-8mm focal length) at 19 frames per second. The petridish was backlit with infrared light at 850 nm (Vishay Semiconductors, VSMY1850x01). To filter out optogenetic illumination, a 715 nm longpass filter was mounted in front of the camera (Thorlabs, FGL715S). Images were captured using the software Point Grey FlyCapture (in trigger mode) as avi files.

*Tracking*. Image analysis was performed using the openCV3.1 library in Python3.5. Briefly, the first frame was subtracted from all other frames, positively labeling only changing pixels, i.e. moving larvae. A threshold was applied to segment the images into binary positively and negatively labeled pixels. Contours were fitted to connected pixels using the function cv2.findContours. Overlapping contours from multiple larvae were discarded based on a fixed area threshold, avoiding potentially incorrect tracking of larvae within close proximity. The center of mass (centroid) was extracted from each contour (cv2.moments) and a rectangle was fitted (cv2.boxPoints). Centroids of contours and lengths of rectangles in each frame provided the measures used for quantification. After the initial detection of a moving larvae, a unique identifier was assigned. This identifier was used to track larvae over time, based on the nearest centroid position in the past frames. Larva crawling tracks were discarded, when one of the following and other criteria applied: contours were missing in > 25 consecutive frames or 28 frames in total; average crawling velocity in the time before illumination was below 0.4 mm/s; many or large jumps in position were detected.

### Electrophysiology assay

For electrophysiology, freshly hatched female flies were collected and fed for 1 d with yeast paste containing 1 mM ATR. Fly preparation, whole-cell patch-clamp electrophysiology, visual and optogenetic stimulation were performed as described previously^6,7,44^, briefly outlined below. We recorded exclusively from tangential cells of the vertical system, i.e. VS cells^22^, tuned to downward motion.

#### Preparation and recording

Flies were tethered with their thorax to a plexiglass holder with bees wax and mounted below a recording chamber to gain access to the back of the head via a small cut-out in the chamber. The cuticle was removed with a hypodermic needle. Under a microscope equipped with polarized light contrast, the glia sheath covering the brain was ruptured using a pulled and collagenase-filled glass capillary^45^ (~5 μm opening) with a combination of mechanical tearing and enzymatic digestion. Whole-cell patch-clamp recordings (current clamp) were obtained from exposed tangential cell bodies with electrodes of 4–7 MΩ resistance. Signals were amplified via a BA-1S bridge amplifier (npi Electronics), low-pass filtered at 3 kHz, and digitized at 10 kHz using an analog/digital converter (PCI-DAS6025; Measurement Computing). Data were acquired in Matlab (R2010b; Mathworks) using the data acquisition toolbox. Normal external solution contained the following (in mM): 103 NaCl, 3 KCl, 5 TES, 10 trehalose, 10 glucose, 3 sucrose, 26 NaHCO_3_, 1 NaH_2_PO_4_, 1.5 CaCl_2_, and 4 MgCl_2_, pH 7.3–7.35, ~280 mOsmol/kg. External solution was carboxygenated (95% O_2_/5% CO_2_) and constantly perfused over the preparation at 2 ml/min. Internal solution, adjusted to pH 7.26 with 1 N KOH, contained the following: 140 K-aspartate, 10 HEPES, 4 Mg-ATP, 0.5 Na-GTP, 1 EGTA, 1 KCl (~265 mOsmol/kg).

#### Visual stimulation

A custom-built LED arena was used for visual stimulation in electrophysiology experiments. The design is based on ref.^46^. The arena covered approx. 170° and 90° in azimuth and elevation, respectively, and allowed refresh rates of 550 Hz and 16 intensity levels^23^. LEDs had an emittance peak at approximately 565 nm and a luminance range from 0 to 51 cd m^−2^. The following stimuli were used in electrophysiology experiments: 1) Moving square wave gratings (spatial wavelength of 24°) at full arena contrast displayed across the entire arena or in a smaller region of the arena (~90° azimuth × ~45° elevation). In between static presentations, the grating was moved in the preferred direction of recorded tangential cells (downward) at a velocity of 24°/s (Fig. 3D) or 19.2°/s (Fig. 3E), corresponding to a temporal frequency of 1 and 0.8 Hz, respectively. 2) An initially static square wave grating (spatial wavelength of 24°) at 30% reduced luminance was presented in a rectangular window of 72° × 72°. Then either ON or OFF edges were moved downward at a velocity of 12°/s (Fig. 3F).

#### Optogenetic stimulation

Optogenetic stimulation was performed as previously described^7^. Light pulses were delivered by a Lambda DG-4 Plus wavelength switcher (Sutter Instrument) with a 300 W Xenon Arc lamp via the epifluorescence light path of the microscope through a 40x/0.8 NA water-immersion objective (LUMPlan FI; Olympus). Light was passed through the following band pass filters: HQ535/30 (Chroma), HQ565/30 (Chroma), FF01-615/20-25 (Semrock). Intensities under the objective were measured with a power meter (Thorlabs PM100D) in air to estimate the irradiance per illuminated area in immersion.

### Optomotor behavior assay

Female flies were selected 1–2 days after eclosion. They were fed with yeast-paste containing 1 mM ATR and kept for two days at 25 °C, 60% humidity on a 12 h dark, 12 h blue light cycle.

#### Tethering flies

Flies were cooled down to 2 °C. The tip of a needle was positioned between head and thorax, slightly tilting the head forwards, allowing direct optogenetic stimulation of the back of the head. Using near-ultraviolet bonding glue (Sinfony Opaque Dentin), which was dried by blue LED light (440 nm, curing light, Guilin Woodpecker Medical Instruments Co., Ltd.), head, thorax and wings were tethered to the needle.

#### Visual stimulation

Behavioral experiments were performed on a locomotion recorder (as described in ref.^30^), using three 144 Hz Monitors (XL2411Z, BenQ). The panels on the monitors were separated from the casing, covered with diffusion foil and vertically arranged into a U-shape. The resulting visual arena has a dimension of 30.5 × 33.5 × 56 cm and a luminance range from 0 to 220 cd m^−2^. The coverage of the fly’s visual field was ±135° horizontally and ±61° vertically with a pixel size smaller than 0.09°. All visual patterns were rendered on a virtual, upright cylinder surrounding the fly, which was positioned in the center of the arena. An additional camera (CM3-U3-13S2C-CS, Point Grey Research) was located on top of the fly, which allowed precise positioning of optogenetic stimulation. All experiments were performed at 34 °C to motivate flies to walk. The following two stimuli were used in the behavioral optomotor assay: 1) Initially static vertical stripes with 10° width and random uniformly sampled intensity were presented only on the left or right side of the fly (+135° to +90° or −90° to −135° in azimuth). After optogenetic stimulation was turned on, the visual pattern was rotated for 2 s with an angular velocity of 80°/s clockwise or counter-clockwise. Subsequently, optogenetic stimulation was turned off - still showing the static visual pattern (Fig. 4C,D). 2) Initially static vertical stripes with 10° width and random uniformly sampled intensity were presented on the left and right side of the fly (resp. +135° to +90° and −90° to −135° in azimuth). As in experiment 1, optogenetic stimulation was turned on shortly after the visual pattern was presented. Subsequently, the visual patterns on both sides were rotated simultaneously front-to-back or back-to-front (angular velocity of 80°/s). Finally, optogenetic stimulation was turned off - still showing the static visual pattern again (Fig. 4E,F).

#### Optogenetic stimulation

Optogenetic illumination was performed using a 565 nm LED (M565F1, Thorlabs), which was coupled into an optical fiber with 105 μm core diameter (M15L01, Thorlabs). A matched achromatic doublet pair (MAP051950-A, Thorlabs) was used to focus the outcoming light from the fiber onto the fly’s head, resulting in a spot of 0.12 mm^2^, similar as described in ref.^5^. Light intensity was measured using a power energy meter (PM100D, Thorlabs).
